# Novel C*ryptosporidium* Genotypes in Sporadic Cryptosporidiosis Cases: First Report of Human Infections with a Cervine Genotype

**DOI:** 10.3201/eid0803.010194

**Published:** 2002-03

**Authors:** Corinne S. L. Ong, Diane L. Eisler, Alireza Alikhani, Vicki W. K. Fung, Joan Tomblin, William R. Bowie, Judith L. Isaac-Renton

**Affiliations:** * University of British Columbia, Vancouver, British Columbia; † BC Centre for Disease Control, Vancouver, British Columbia; ‡ BC Biomedical Laboratories Ltd., Surrey, British Columbia, Canada.

**Keywords:** *Cryptosporidium*, cryptosporidiosis, molecular, genotype, polymerase chain reaction, restriction fragment length polymorphism, 18S rRNA, sequencing

## Abstract

In this study, we genotyped parasites from the fecal specimens of sporadic cryptosporidiosis cases in British Columbia from 1995 to 1999. Genotyping was conducted by polymerase chain amplification of the internal transcribed spacer region, a hypervariable region in the 18S rRNA gene and the *Cryptosporidium* oocyst wall protein gene. Subsequent analysis was by restriction fragment length polymorphism and DNA sequencing. We identified two new *Cryptosporidium* genotypes in humans. One of these genotypes has been found recently in deer in New York state. The other genotype has not been identified in humans or animals. These results have important implications for drinking water quality strategies, especially for communities that obtain drinking water supplies from surface sources located in forested regions with deer populations.

In recent studies of cryptosporidiosis cases in North America, South America, Europe, and Australia, various polymorphic gene loci were used to show that two major genotypes of *Cryptosporidium parvum* occur in humans (*1*–*10*). Genotype 1, or the human genotype of *C. parvum*, has been isolated almost exclusively from humans and associated mainly with anthroponotic (human-to-human) transmission cycles (*1*). Experiments to infect animals such as cattle and mice with the human genotype have been unsuccessful, and the only in vivo model that exists for this genotype is a gnotobiotic piglet model (*11*). So far, the only animals reported to be infected with genotype 1 *C. parvum* are a monkey in the United States (*5*) and a dugong (*Dugong dugon*) in Australia (*12*). In contrast, genotype 2 or the calf genotype of *C. parvum* has been isolated from both human and bovine hosts, as well as other livestock and wild animals such as sheep, goats, and deer. Genotype 2 has been associated with zoonotic (animal-to-human) transmission cycles.

Other genotypes of *C. parvum* are found in animals,. including the dog, mouse, bear, pig, deer, and marsupial genotypes of *C. parvum,* which have been differentiated by sequence polymorphisms in the small subunit ribosomal RNA (*13*–*15*), the acetyl CoA synthetase (*15*), and heat shock protein 70 (*16*), as well as the *Cryptosporidium* oocyst wall protein (COWP) (*17*) genes, and named after the animals from which they were derived. Of these variant *C. parvum* genotypes, three human infections with the dog genotype have been reported—in an HIV patient (*4*), two Peruvian children (*6*), and a child in England (*18*). Aside from *C. parvum*, nine other *Cryptosporidium* species are recognized: *C. felis* (cat), *C. muris* (rodent), *C. andersoni* (cattle), *C. wrairi* (guinea pig), *C. baileyi* (bird), *C. meleagridis* (bird), *C. serpentis* (reptile), *C. surophilum* (lizard), and *C. nasorum* (fish). Although previously thought to be host specific, these other *Cryptosporidium* species have been associated with a few reports of human infections. *C. felis* (*4*,*18*,*19*) and *C. meleagridis* (*19*) have been found in immunocompromised persons. In addition, *C. felis* (*6*,*18*), *C. meleagridis* (*6*), and possibly *C. muris* (*20*) infections have been reported in children.

In this study, we genotyped parasites from the fecal specimens of sporadic cryptosporidiosis cases in British Columbia (BC). Genotyping was conducted by polymerase chain reaction (PCR) amplification of the internal transcribed spacer region, a hypervariable region in the 18S rRNA gene (*4*) and the COWP gene, (*21*). Subsequent analysis was by restriction fragment length polymorphism (RFLP) and DNA sequencing. We identified two new *Cryptosporidium* genotypes in humans. One of these genotypes has been found recently in deer in New York State (*14*). The other genotype has not been identified in humans or animals.

## Materials and Methods

### Cryptosporidiosis Cases and Community Information

*C. parvum* oocysts were isolated from patients in the Greater Vancouver and Fraser Valley Regional Districts of British Columbia over a 5-year period from 1995 to 1999. Demographic data on this geographic area have been described (*2*). Fecal specimens were collected from patients diagnosed with clinical symptoms consistent with cryptosporidiosis. *Cryptosporidium* oocysts were identified in stool specimens by standard concentration methods, acid-fast staining, and microscopy by the diagnostic parasitology laboratories to which the specimens were submitted. Oocyst-containing specimens were preserved in potassium dichromate solution (2.5% w/v) within 7 days of reception and stored at 4°C. The study was conducted retrospectively on specimens that were coded without personal identifiers. Informed consent from subjects was obtained.

### Genomic DNA Extraction

Resuspended stool specimens were strained through cheesecloth. Potassium dichromate was removed by washing the sedimented filtrate 3 times in distilled water . Lipids were then extracted by using ethyl acetate as described (*2*). *Cryptosporidium* oocysts were disrupted by repeated freezing in a dry ice-ethanol bath and thawing in a boiling water bath in a 20% w/v suspension of Chelex-100 (BioRad Laboratories, Hercules, CA) as described (*2*)*.* The DNA extracts were stored at –20°C.

### PCR Amplification of *C. parvum* Oocyst DNA

Genomic DNA extracts from oocysts were centrifuged at 11,000 rpm (9,000 x *g*) for 20 minutes at 4^°^C and the supernatants used as template DNA for PCR. The PCR reaction was carried out as described (*2*) by using the forward primer, cry7, and the reverse primer CP5.8R to amplify the entire internal transcribed spacer 1 (ITS1) region, resulting in a 600-bp product. The amplification procedure using the CPBDIAGF/CPBDIAGR primer pair described by Pieniazek et al*.* (*4*) was used to amplify the hypervariable region of the 18S rRNA gene, and the CRY-9/CRY-15 primer pair described by Patel et al. (*21*) was used for the COWP gene.

In addition, genomic DNA prepared from oocysts that had either been characterized in previous studies (*1*,*2*) or were isolated from well-defined sources were included as known genotype controls. Genotype 2 controls included one bovine isolate from a purified batch of Iowa strain oocysts that had been passaged in calves at the University of Arizona; two human isolates from 1996 Cranbrook and 1998 Chilliwack outbreak cases, where animals infected with cryptosporidiosis were found in the watershed area ([*2*] and Ong et al., unpub. data); and five other human isolates derived from sporadic cases in British Columbia that have been described in a previous study as *C. parvum* genotype 2 isolates (*2*). Genotype 1 controls included seven isolates from sporadic cases and one isolate from a 1996 Kelowna outbreak case (*2*), all identified previously as *C. parvum* genotype 1 isolates (*2*). Deionized water and a culture of a nonpathogenic strain of *Escherichia coli* were used as negative controls.

### RFLP Analyses of PCR Products

PCR products were purified by using QIAquick spin columns (Qiagen, Mississauga, ON) according to the manufacturer’s instructions before digestion with the restriction endonucleases *Mse* I (New England BioLabs, Mississauga, ON) for the ITS1 locus and *Rsa* I (New England BioLabs) for the COWP gene. Two units of enzyme were added to a final volume of 20 μL containing 15 μL of PCR product and the appropriate dilution of the manufacturer’s recommended buffer, and then incubated overnight at 37°C. Restriction fragments were then separated on Metaphor FMC agarose gels (3% for *Mse* I digests of ITS1 products and 3.2% for *Rsa* I digests of COWP products) (Mandel Scientific, Guelph, ON) and stained with ethidium bromide; the patterns were visualized with a UV transilluminator. DNA band sizes were analyzed by using the ProRFLP program version 2.38 (DNA ProScan Inc., Nashville, TN).

### DNA Sequencing and Analyses

PCR products from the variable 18S rRNA and COWP gene loci were cleaned by spin column purification using the QIAquick PCR Purification kits (Qiagen). Sequencing reactions were conducted in both directions, i.e., from the 5’ and 3’-ends using the ABI Prism BigDye Terminator Cycle Sequencing Kit (Applied Biosystems, Foster City, CA), and analyzed on an ABI 310 automated DNA analyzer (Applied Biosystems).

Overlapping bidirectional sequences were assembled by using the SeqManII (DNASTAR Inc., Madison WI) sequence analysis software. Consensus sequences obtained were aligned by using the ClustalX program (*22*), which was also used for calculating the phylogenetic tree by the neighbor-joining method with 1,000 replicates for bootstrap values. Published 18S rRNA gene reference sequences included in the multiple sequence alignment are listed below with their corresponding accession numbers: AF093491 (*23*) and AF087575 (*4*) for *C. parvum* genotype 1 human isolates; AF112569 (*13*) for a *C. parvum* simian isolate; AF087576 (*4*) and AF093490 genotype 2 isolates from a human and a bovine source, respectively; AF087574 (*4*) and AF112576 (13) for *C. parvum* “dog” genotype isolates from a human and a canine source, respectively; AF115377 (*13*), AF247535 (*24*), and AF112571 (*13*) for *C. parvum* pig, bear, and mouse genotype isolates, respectively; AF297511 (*14*), AF297512 (*14*), and AF297515 (*14*) for *C. parvum* “deer” genotype isolates; AF297503 (*14*) for a *C. parvum* muskrat isolate; AF087577 (*4*) and AF112575 (*13*) for *C. felis* from a human and a feline source, respectively; AF115378 (*13*), AF093498 (*23*), AF093496 (*23*), AF112574 (*13*), AF093495 (*23*), and AF093499 (*23*) for *C. wrairi*, *C. muris*, *C. andersoni*, *C. meleagridis*, *C. baileyi*, and *C. serpentis*. The phylogenetic tree was displayed visually by using TreeView (*25*). The *C. muris*, *C. andersoni,* and *C. serpentis* sequences were used in the outgroup, and the tree was rooted with this outgroup.

The 18S rRNA and COWP gene sequences of the 11 patient isolates listed in the table have been submitted to GenBank and assigned accession numbers AY030084 to AY030093 and AF411631 to AF411633. The BLAST server (http://www.ncbi.nlm.nih.gov/BLAST/) was used for DNA databases searches.

## Results

**Figure 1:**
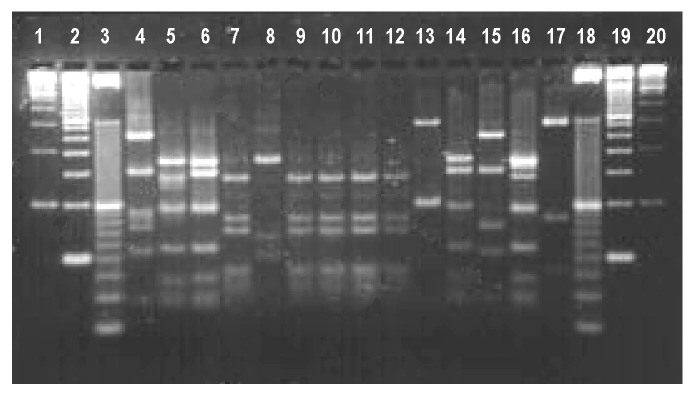
Restriction profiles obtained after digestion of polymerase chain reaction products from the ITS1 locus with *Mse* I. lanes 1- 3-, 100-, 50-, and 10-bp ladder molecular weight markers; lanes 4 and 15, bovine genotype 2 isolates; lanes 5, 6, 14, and 16, human genotype 1 isolates including DE340 (lane 14); lanes 7 and 9–12, cervine genotype isolates including MH205 (lane 7), TK320 (lane 10), and DE302 (lane 11); lane 8, *Cryptosporidium meleagridis* isolate CS33; lanes 13 and 17, other novel genotype isolates such as VF383 (lane 13) and TK348 (lane 17)

*Cryptosporidium* oocysts were isolated from fecal specimens of 150 sporadic cryptosporidiosis cases. Two characteristic restriction profiles were obtained for *Mse* I digests of the 600-bp ITS1 products (Figure 1). The first type of restriction profile (Figure 1, lanes 4 and 15) showed five major bands at approximately 270, 160, 90, 75, and 55 bp. The bovine isolate, patient isolates from the 1996 Cranbrook and the 1998 Chilliwack outbreaks, and 29 (19%) isolates from sporadic cases had this restriction profile. Based on results from previous molecular characterization of a number of these isolates (*1*,*2*), this restriction profile was considered to be the *C. parvum* genotype 2 restriction pattern. The second restriction pattern (Figure 1, lanes 5, 6, 14, and 16) with six major bands around 185, 150, 100, 60, 40, and 30 bp was obtained from isolates of 108 (72%) sporadic cases and the one patient from the 1996 Kelowna outbreak. This restriction profile was considered to be the *C. parvum* genotype 1 profile, based on results from previous molecular analyses on the other seven genotype 1 isolates that were included as human genotype controls (*2*).

Restriction profiles with varying patterns (Figure 1, lanes 7, 9 to 13 and 17) were obtained from 13 (9%) other human isolates. Of these, nine (6%) isolates had identical restriction profiles (Figure 1, lanes 7 and 9 to 12) with five major bands at 150, 85, 70, 45, and 35 bp and were designated cervine genotype isolates. The other four isolates had unique restriction profiles and could be split into two groups of two isolates, based on the similarity of banding patterns. These were CS33 (Figure 1, lane 8) and MH222 (data not shown), which both had restriction fragments at 175 and 50 bp and additional variant bands at 65 and 70 bp, respectively. The remaining two isolates VF383 had bands at 315 and 105 bp (Figure 1, lane 13) and TK348 had bands at 325 and 85bp (Figure 1, lane 17), respectively.

**Figure 2:**
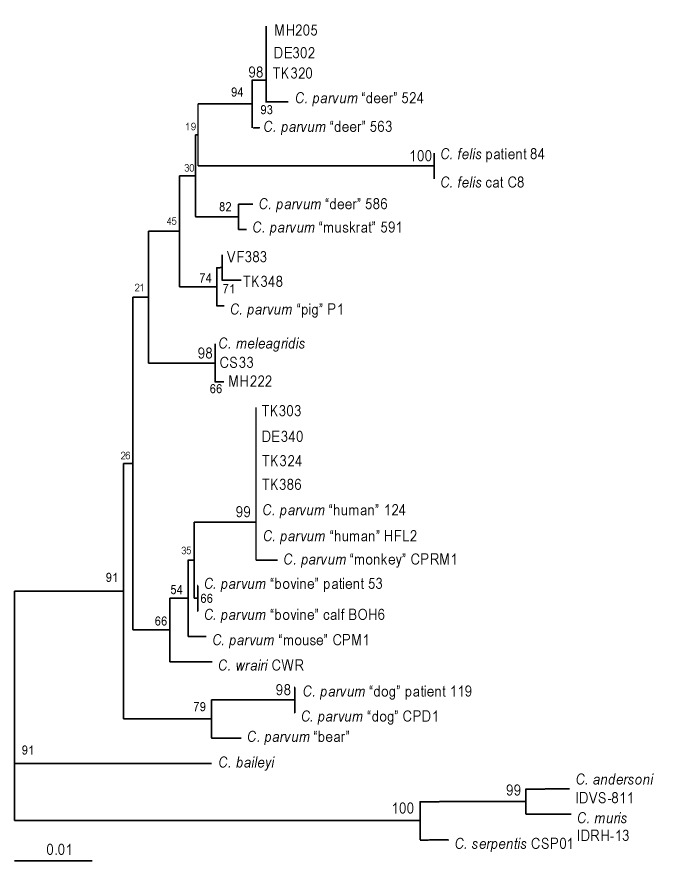
Phylogenetic relationship of isolates from sporadic cases with reference 18S rRNA gene sequences from various *Cryptosporidium* species and genotypes. Bootstrap values that are >95% are shown in larger font. Bar = 0.01 substitution per site.

To identify the *Cryptosporidium* species and genotype of isolates with variant restriction profiles, sequencing of a polymorphic locus on the 18S rRNA gene was carried out. Eleven isolates were selected based on their ITS1 restriction patterns. These included one isolate (TK386) with a characteristic human genotype 1 restriction profile, three isolates (TK324, TK303, DE340) with patterns similar to the human genotype 1 restriction profile but with one restriction fragment shifted slightly in molecular size (e.g., Figure 1, lane 14), three cervine genotype isolates (MH205, TK320, DE302; Figure 1, lanes 7, 10, and 11), and four isolates (CS33, MH222, VF383, TK348) with unique variant profiles (Table). Comparison of the 18S rRNA gene sequences of these isolates with 22 other published reference sequences, derived from a variety of human and animal *Cryptosporidium* isolates by using multiple sequence alignment and phylogenetic analysis (Figure 2), showed that the 11 isolates fell into four main groups. The first group consisted of four isolates (TK386, TK324, TK303, and DE340) that had restriction profiles identical or similar to the characteristic human genotype 1 pattern and all human genotype 1 reference isolates. All human isolates in the genotype 1 group had the characteristic polyT repeat sequence reported previously (*10*,*26*) between positions nt 686 and 698. The second group consisted of three human isolates (TK320, DE302, and MH205) and two cervine genotype isolates (Figure 2). The sequences of these three human isolates in the hypervariable 18S rRNA region were identical to that of a genotype 3 deer isolate described by Perz and Le Blancq (*14*). The third group consisted of two isolates (VF383 and TK348) and a pig genotype isolate (Figure 2). Sequences between the two human isolates were variant from the pig genotype sequence at only two different nucleotide positions between nt 686 and 698. The fourth group consisted of two human isolates (MH222 and CS33) and a *C. meleagridis* isolate (Figure 2).

**Table:** Analysis of *Cryptosporidium* isolates collected from 1995 to 1999 from sporadic cases in British Columbia, Canada

Selected isolates	Genotype	RLFP loci	Genes sequenced
TK386	Human	ITS1	18S
TK303	Human	ITS1	18S
TK324	Human	ITS1	18S, COWP
DE340	Human	ITS1	18S
MH205	Cervine	ITS1, COWP	18S, COWP
TK320	Cervine	ITS1	18S, COWP
DE302	Cervine	ITS1	18S
MH222	C. meleagridis	ITS1	18S
CS33	C. meleagridis	ITS1, COWP	18S
VF383	Other novel	ITS1	18S
TK348	Other novel	ITS1	18S

RFLP = restriction fragment length polymorphism; ITS1 = internal transcribed spacer 1; COWP = *Cryptosporidium* oocyst wall protein.

**Figure 3:**
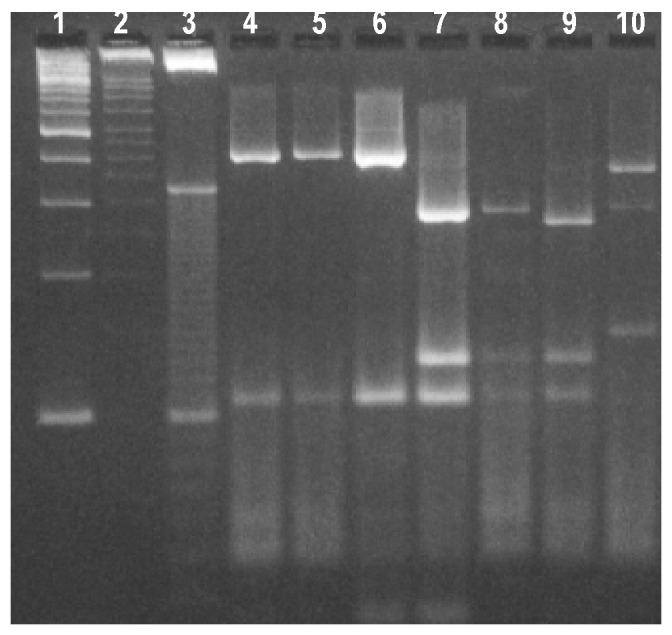
Restriction profiles obtained after digestion of polymerase chain reaction products from the *Cryptosporidium* oocyst wall protein locus with *Rsa* I. Lanes 1- 3-, 100-, 50-, and 10-bp ladder molecular weight markers; lanes 4 and 6, bovine genotype 2 isolates; lanes 7 and 8, human genotype 1 isolates; lane 9, cervine genotype isolate MH205; and lane 10, *C. meleagridis* isolate CS33

Twenty-five sporadic isolates were also characterized by using a second locus on the COWP gene. *Rsa* I digests of the 550-bp PCR products (Figure 3) also showed the same dimorphism as the ITS1 locus with two predominant restriction patterns. Of these, 10 (40%) isolates had fragments at approximately 285, 125, 105, and 35 bp, which were characteristic for genotype 1 isolates (Figure 3, lanes 7 and 8). Another six (24%) isolates had the genotype 2 restriction profile with fragments at 410, 105, and 35 bp (Figure 3, Lanes 4 to 6). One isolate (CS33) had a variant restriction profile (Figure 3, Lane 10) with bands at approximately 370, 290, and 150 bp, which were similar in size to fragments reported for a *C. meleagridis* isolate (*26*). The 18S rRNA gene sequence of this isolate (CS33) was identical to that of *C. meleagridis*. The other isolate (MH205), had a restriction profile that was identical to those obtained from genotype 1 isolates (Figure 3, lane 9). This isolate had an identical ITS1 restriction profile with eight other sporadic human isolates and an 18S rRNA gene sequence that grouped with deer genotype 3 isolates from New York (Figure 2). The COWP gene sequences of MH205 and another cervine genotype isolate TK320 were determined to be identical and novel, sharing only 90% and 91% identity with the COWP gene sequences of the human (AF248741) and bovine (AF248743) alleles, respectively. BLAST analysis showed most alignment (92% identity) with a pig COWP gene sequence (AF266270) (*17*). However, the cervine allele had identical *Rsa*I restriction sites to the human allele at nt 34, 228, 512, and 618, whereas the bovine allele lacked the site at nt 228. The RFLP patterns could not be determined for the remaining seven isolates as insufficient PCR product was obtained. Over half (51%) of the isolates in this study were derived from pediatric patients <10 years of age, which accounted for seven of the nine cervine genotype infections as well as the two *C. meleagridis* infections.

## Discussion

This study describes the discovery of the first zoonotic infections in humans with a novel cervine *Cryptosporidium* *parvum* genotype. Perz and LeBlancq (*14*) described this genotype recently after characterizing 111 *Cryptosporidium* isolates from wildlife in New York state. Those researchers did not detect human infections with this genotype in cryptosporidiosis cases in New York City. Other molecular epidemiologic studies in England (*8*,*18*) of 1,705 cases also did not identify cervine genotype infections, although rare zoonotic infections in humans with the dog genotype of *C. parvum* as well as other *Cryptosporidium* species such as *C. felis* and *C. meleagridis* were found (*27*). It is possible that cervine genotype infections in humans were not identified because the novel deer genotype had not been reported at the time of the study. As well, the PCR/RFLP profile of the COWP gene from the cervine genotype isolate was identical to that obtained from human genotype 1 isolates. Sequencing of the COWP gene from two cervine genotype isolates confirmed that the human and cervine alleles had identical *Rsa*I restriction sites. Therefore, RFLP analysis using this endonuclease could not differentiate between isolates with these two genotypes.

Xiao et al. (*28*) have also found this novel cervine genotype in storm water samples collected from a stream in the watershed area of New York State that contributes to the New York City water supply. The transmission of cryptosporidiosis from wildlife to humans in British Columbia is not surprising as many communities are supplied with unfiltered drinking water drawn from surface sources where *Cryptosporidium* spp. oocysts have been detected (*29*). Many of these watersheds are situated in remote forested areas, where wildlife such as deer are present in abundance. Deer with cryptosporidiosis infections have been identified in these watersheds (Ong et al., unpub. data). Therefore, to have as many as 6% of sporadic cases infected with this novel deer genotype is not an unexpected finding.

The ITS1 and 18S rRNA genes are reportedly multicopy genes with four copies of the Type A and one copy of the Type B rDNA units per haploid genome (*30*). The sequence divergence found between Type A and Type B units in the ITS1 region was a concern to us initially, as we first characterized the isolates using this locus before this report. However, Morgan et al*.* (*31*), who conducted a similar PCR-RFLP analysis of the ITS1 region, found that the restriction profiles were specific for different *C. parvum* genotypes. This study also indicated that intraorganism variation caused by the difference between Type A and Type B rDNA units may not be such a problem. Using primers to amplify the Type B unit, Morgan et al. (*31*) found that the Type A unit was amplified preferentially for human genotype isolates. To confirm that the observed variation in the ITS1 RLFP patterns was not due to heterogeneous products amplified from different copies of rDNA, further characterization of a select number of sporadic isolates was performed with the 18S rRNA as well as the COWP genes. Results from these additional analyses showed that isolates with distinctly different ITS1 RFLP patterns had different COWP RFLP patterns as well as 18S and COWP gene sequences. Therefore, ITS1 RFLP was useful for generating characteristic fingerprints that could distinguish between different *C. parvum* genotypes and *Cryptosporidium* species.

Using this method of genotyping, we were able to detect two new genotypes of *C. parvum* that had not been reported previously. Nine isolates (including three, MH205, TK320, and DE302, which had been characterized at the 18S locus; and two, MH201 and TK320, which had been characterized at the COWP locus) had the cervine genotype. Two other isolates (VF383 and TK348) had novel genotypes that were most closely related to a pig genotype isolate from Switzerland (*13*). These results have important implications for drinking water quality strategies, especially for communities that obtain drinking water supplies from surface sources located in forested regions with deer populations.
